# The use of HRT containing transdermal estradiol in women with a personal history of venous or arterial thromboembolism: A consecutive series of 115 cases

**DOI:** 10.1177/20533691251369205

**Published:** 2025-08-22

**Authors:** Phoebe Howells, Sadiya Hussain, Emily Hulme, Kugajeevan Vigneswaran, Samar Zia, Mohsen Hassan, Roopen Arya, Haitham Hamoda

**Affiliations:** 1 156446 Princess Royal University Hospital, Orpington, UK; 2 111990 King’s College Hospital, London, UK; 3 King’s College Hospital Fertility, London, UK

**Keywords:** Menopause, HRT, estrogen, postmenopause

## Abstract

**Objective:**

To assess the risk of further thrombosis with using HRT containing transdermal estradiol in women with a personal history of venous thromboembolism (VTE) or arterial thromboembolism (ATE).

**Methods:**

We undertook analysis of patients with a history of VTE/ATE who had been seen in the menopause clinic at King’s College Hospital. They were followed up for a minimum of a 12 months period to assess for recurrence of VTE/ATE.

**Results:**

The study included 115 patients with a personal history of VTE/ATE who had used HRT for over a year. 81% had a history of a VTE, 19% had suffered from an ATE. Patients had taken HRT containing transdermal estradiol +/− micronised progesterone or Mirena coil or combined transdermal HRT for a range of 1–20 years, with an average duration of 3 years. 11 patients had switched from oral HRT to transdermal HRT after visiting the clinic. All patients had been referred to a haematologist with 20% of patients requiring life-long anticoagulation based on their medcial background history. 64% of the patients had documented thrombophilia results with 42% of those being positive for a thrombophilia. 39/115 patients required a higher dose of estrogen, taking either more than two pumps of gel/spray or >50 micrograms in the form of a patch. Significantly on follow up (up to 12 months or more), none of the patients had a recurrence of a thromboembolism within 12 months of starting HRT containing transdermal estradiol.

**Conclusion:**

To our knowledge, this is the first study assessing the use of HRT containing transdermal estradiol in women with a personal history of VTE/ATE. None of the patients assessed had a thromboembolic event within 12 months of follow up. This study will enable more clinicians to consider prescribing HRT in patients with a personal history of VTE/ATE and higher patient uptake, after individualised risk-assessments.

## Purpose

Several observational studies have shown that, unlike oral estradiol, HRT containing transdermal estradiol has a neutral effect on the risk of VTE and stroke and does not significantly increase the risk of VTE or stroke beyond an individual predisposition. Laboratory studies support this neutral effect on thrombotic risk and show that transdermal estradiol does not impact coagulation or inflammatory markers.

As a result, national and international guidance advises that women with known VTE/stroke risk factors may safely consider HRT using transdermal estradiol. However, there is limited evidence assessing outcomes in women at the highest risk, namely, those with a personal history of thrombosis.

We present what to our knowledge is the first case series of women with a personal history of VTE or stroke who have taken HRT containing transdermal estradiol.

## Introduction

Navigating through menopause marks a significant life change, often accompanied by various troublesome symptoms. Vasomotor symptoms, cognitive changes, and many other problems are all challenging manifestations of the menopausal transition. Hormone Replacement Therapy (HRT) is an important option for many women and can significantly improve quality of life.^1,2^ While estrogen replacement, with or without progestogen and/or testosterone, offers the prospect of benefit, barriers persist in accessing treatment. Medical professionals often struggle with uncertainty, weighing the risks against the benefits, particularly in high-risk scenarios, and may as a result be advising women against HRT, due to a particular co-morbidity.^3^

Our aim in this study was to assess the risk of further thrombosis when using HRT containing transdermal estradiol in women with a personal history of venous thromboembolism (VTE) or arterial thromboembolism (ATE).

The Women’s Health Initiative (WHI) as well as other randomised controlled trials reported that HRT containing oral estrogen increased the risk of VTE including Pulmonary Embolism (PE) and Deep Vein Thrombosis (DVT) as well as the risk of stroke. Following these findings, guidelines were updated to reflect the potential risks and healthcare providers began to re-evaluate individual risk factors when prescribing HRT for their patients. An increased emphasis on non-hormonal alternatives for managing symptoms in these high-risk women followed and women often manage debilitating symptoms conservatively.^3^

There are numerous risk factors to consider that can increase an individual’s inherent risk of VTE, including advanced age, elevated body mass index (BMI), personal or family history of VTE, or other factors such as known thrombophilia. While these intrinsic factors might influence risk, recent evidence emphasises that the primary determinant of the thrombotic risk associated with HRT intake, is the route of administration of estradiol with HRT.^1^

When considering HRT for patients with a history of arterial thromboembolism, in particular stroke, evidence from many observational studies have shown that HRT preparations containing transdermal estradiol are unlikely to increase the risk of stroke compared to non-users. It is also associated with a lower risk of stroke when compared to oral HRT.^4,5^ The safety of transdermal estradiol with regard to thrombotic risk is strongly supported by laboratory studies examining markers of coagulation and fibrinolysis as well as thrombin generation which is a global coagulation assay. These various laboratory studies have consistently shown that transdermal estradiol has a neutral effect and that is unaltered by increasing doses within standard prescribing doses.^6^ The British Menopause Society (BMS) advice is therefore to consider HRT containing transdermal in those who are high risk of VTE/ATE or over the age of 60.^1,7,8^

Similarly, the advice for those with hereditary thrombophilia such as the Factor V Leiden and Prothrombin gene mutations, is to consider HRT containing transdermal estradiol as this is unlikely to increase the risk of VTE/ATE above background risk.^5,9^ Whilst testing for thrombophilia is only advised on a selective basis in those with risk factors such as family history, a positive result would trigger a consultation with a haematologist and the advice is that HRT containing transdermal estradiol is unlikely to increase their VTE risk above their own background risk.^3^

## Method

Consecutive cases with a personal history of venous or arterial thromboembolism who have taken HRT containing transdermal estradiol with a minimum follow-up period of 12 months at a tertiary referral menopause specialist service were included in this review. After assessment and benefit/risk discussion and in discussion with the local haematology team, those who were suitable and keen to proceed with HRT were prescribed HRT containing transdermal estradiol.

We used a search engine COGSTACK to identify these patients who had a history of VTE or ATE and reviewed those that had been on HRT for a minimum of 1 year from 2015 to 2020. These patients were interviewed using a structured questionnaire, in particular to elicit occurrence of recurrent thrombosis.

## Results

We included a cohort of 115 patients who had a history of VTE/ATE and had used HRT and who were followed up for a minimum of 1 year during this period. Included women had an age range of 29–58 years old with a median age of 53 years old. Their BMI ranged from 16 to 43 kg/m^2^ with 68/115 (60%) having a BMI <30 kg/m^2^, 35/115 (30%) with a BMI 30–35 kg/m^2^ and 12/115 (10%) with a BMI >35 kg/m^2^.

The majority of patients were Caucasian 84/115 (73%) with 17/115 (15%) being of Black African or Afro-Caribbean descent as seen in Table 1.

**Table 1.** Ethnicity of patients included in the study.

Ethnicity	Number of patients (percentage %); total number of patients = 115
Caucasian	84 (73)
Black African or Afro-Caribbean	17 (15)
Other European	9 (7)
Asian	3 (3)
Latin America	2 (2)

33/115 (28%) were nulligravida, 18/115 (15%) were primigravida and 75/115 (65%) had a parity of one or more. 7/115 (6%) were of unknown parity. 93/115 (80%) were non-smokers with 11/115 (9%) and 8/115 (7%) being ex-smokers or current smokers, respectively.

13/115 (11%) had a medical history of either current or past malignancy. 12/115 (10%) of these had been haematological malignancies with one patient having had thyroid cancer. 34/115 (30%) had a positive family history of a first-degree relative having had a VTE/ATE. Although thrombophilia testing is not routinely required, as it is unlikely to alter the management plan, 64% of the patients had documented results with 42% of those being positive for a thrombophilia.

94/115 (81%) had a personal history of VTE, 21/115 (19%) had suffered from an ATE. Table 2 shows the number of cases of each type of thromboembolism.

**Table 2.** Type of thromboembolism.

Type of thromboembolism	Number of patients
DVT	50
PE	28
DVT + PE	9
Portal vein thrombosis	4
Jugular/subclavian vein thrombosis	11
PICC associated thrombosis	1
Arterial ischaemic stroke	18
Femoral artery thrombosis	1
Retinal artery thrombosis	1
Right atrial thrombosis	2
Recurrent VTE/ATE	11

42/94 patients had a known provoking factor for their VTE with 10/42 being linked to a recent long-haul flight as shown in Table 3.

**Table 3.** Provoking factors for venous thromboembolism.

Potential cause of VTE	Number of patients; n = 94
Flight	10
Postoperative	5
Pregnancy	5
Post line insertion	5
Postnatal	4
COCP	4
Hospital admission	2
Post ankle fracture	2
High BMI	2
Drug abuse	1
Oral HRT	1
Medical post UC flare	1
No obvious provoking factor	52

All patients were referred to/discussed with the haematology team before starting HRT. This was documented in the correspondence clinic letters in 89/115 (77%). There was no documented evidence of a case where the haematologist did not agree that it was safe. 20 percent of patients were already receiving life-long anticoagulation in view of an increased background risk for further VTE.

The decision regarding the need for long-term anticoagulation was based on existing risk factors and the commencement of HRT did not alter this. Patients who required long-term anticoagulation did so regardless to whether they opted to take HRT or not and the addition of HRT with transdermal estradiol did not result in the need for additional anticoagulation.

Patients had taken HRT containing transdermal estradiol for a range of 1–20 years, with an average duration of 3 years. 11/115 (10%) patients had switched from oral HRT to transdermal HRT after visiting the clinic.

During their course of treatment 39/115 (34%) of patients required an increase in their dose of their estrogen, taking either more than two measures of gel/spray or >50 micrograms in the form of a patch.

None of the patients assessed within this review had a recurrence of thromboembolism after starting transdermal estrogen within the 12 months follow-up period.

## Discussion

This consecutive series which reports the use of HRT containing transdermal estradiol in women with a personal history of venous or arterial thromboembolism showed no recurrence of thrombotic events within 12 months of follow up. This suggests that HRT containing transdermal estradiol can be safely considered in women with a history of thromboembolism.

Large observational data have shown that the risk of venous or arterial thromboembolism is unlikely to increase above background risk in women taking HRT containing transdermal estradiol. However, there is lack of reported outcomes from women with a personal history of thrombosis who are subsequently exposed to transdermal estradiol.^1^

Whilst combined contraceptives which contain ethinylestradiol exhibit increased prothrombotic effects whether in transdermal or oral form, thereby conveying similar increased risk for VTE, this is not the same in relation to the route of administration of estradiol with HRT, where transdermal preparations contain estradiol.^10^

The connection between HRT containing oral estradiol and VTE has been thoroughly investigated and is widely acknowledged. Published literature consistently indicates a 2- to 4-fold higher risk of VTE, especially within the initial year of starting treatment, with the greatest risk occurring in the first 6–12 months. This risk gradually declines over subsequent periods.^1,8^

The risk of VTE with HRT containing oral estradiol is also dependent on the dosage and type of estrogen, where conjugated equine estrogens pose a greater thrombotic risk compared to estradiol. Similarly, the type of progestogen in oral HRT is also likely to play a significant role in VTE risk, with studies suggesting that micronised progesterone and dydrogesterone are associated with a lower risk compared to other synthetic progestogens such as norethisterone and medroxyprogesterone.^1,11^

It is however now evident that estradiol administered transdermally within HRT does not elevate VTE risk beyond that of non-users.^1,8^

To understand the safety of transdermal preparations in high-risk VTE patients, it is key to understand the mechanism by which hormonal intake acts on the coagulation pathway. Transdermal preparations have the benefit of by-passing first-pass metabolism by the liver and consequently the effect on coagulation pathways is much less. Even with the by-passing, the effects of transdermal hormonal preparations on VTE are dependent on the type of estrogen. As described above, transdermal HRT contains estradiol which is physiologically similar to ovarian estrogen and does not significantly increase clotting factors transdermally. Transdermal combined contraceptives contain the synthetic estrogen ethinylestradiol, this promotes a prothrombotic state even though it bypasses the first-pass metabolism of the liver, raising the risk of VTE.^12^

The physiological changes in the coagulation cascade associated with intake of oral estrogens are well described, including alterations in levels of procoagulant factors such as fibrinogen, prothrombin, factors VII, VIII, and X, alongside a reduction in natural anticoagulants such as anti-thrombin and protein S. These changes predispose individuals to a heightened risk of thrombosis.^13,14^

Thrombin generation is a useful overall marker for the coagulation pathway activation and a surrogate marker for VTE risk.^6^ A prospective cohort study revealed that oral HRT led to the rapid and elevated production of thrombin compared to HRT containing transdermal estradiol. The latter resulted in thrombin levels similar to those observed in non-HRT users. Additionally, estrone levels were markedly higher in oral HRT compared to transdermal HRT and were associated with peak thrombin generation. Eestrone levels are known to be a more potent inducer of liver protein synthesis. Thrombin generation is stimulated by high estrone levels following intake of HRT containing oral but not transdermal estradiol (Morris 2023).^3^ Other studies have shown that HRT containing oral estradiol consistently increased activated protein C resistance, but transdermal estradiol had little or no effect on the coagulation cascade or fibrinolysis.^15,16^

In the current study we evaluated outcomes in women confirmed to be at high risk due to their personal history of thrombosis who were exposed to HRT containing transdermal estradiol. None experienced recurrence whilst taking transdermal HRT preparations within a 12 months period. The subjects included a substantial proportion of women found to have thrombophilic abnormalities, which adds further reassurance regarding the safety of transdermal HRT. This was very much a real-world population with diverse ethnicities and 40% of the women being classed as overweight or obese. Half of those with VTE had unprovoked events; these are usually considered in the higher risk category for recurrence. Standard advice was given to all patients regarding thromboprophylaxis during high-risk situations such as hospitalisation, long-haul flights and other potentially provoking events. It is important for all these cases to be at least discussed with a haematologist before starting HRT, although those with significant risk factors should be referred for review.

## Limitations

Due to the relatively small sample size and observational nature of the study, these findings require further assessment in larger controlled studies. There might also be selection bias since these women were managed in a specialist hospital clinic setting.

In conclusion our findings suggest that women with previous thrombotic events can safely receive HRT containing transdermal estradiol and this is unlikely to increase the risk of further thrombotic events above their background risk. The highest-risk women might already be on anticoagulation and there is evidence that this is effective in mitigating hormone-related thrombotic risk.^17^

This study provides reassuring evidence for the safety of HRT containing transdermal estradiol in women with a personal history of VTE/ATE. Our findings should enable safe prescribing of HRT in high-risk women after an individualised risk assessment and empower such patients to receive appropriate advice and therapy. Further studies and sharing of experience of transdermal HRT use in this setting are desirable and should provide additional reassurance.
